# {*N*′-[(*E*)-(5-Bromo-2-oxidophen­yl)(phen­yl)methyl­ene]benzohydrazidato}pyridine­copper(II)

**DOI:** 10.1107/S1600536809030463

**Published:** 2009-08-08

**Authors:** Chang-Zheng Zheng, Chang-You Ji, Xiu-Li Chang, Chao-Hua Zhang, Yang-Guang Xiang

**Affiliations:** a College of Environmental and Chemical Engineering, Xi’an Polytechnic University, Xi’an, Shaanxi 710048, People’s Republic of China; b School of Chemistry & Pharmaceutical Engineering, Sichuan University of Science & Engineering, Zigong, Sichuan 643000, People’s Republic of China; c Liaoning Key Laboratory of Applied Chemistry, Institute of Superfine Chemicals, Bohai University, Jinzhou,121000, People’s Republic of China

## Abstract

The asymmetric unit of title complex, [Cu(C_20_H_13_BrN_2_O_2_)(C_5_H_5_N)], contains two independent mol­ecules. In each mol­ecule, the central Cu^II^ atom has a square-planar environment formed by the tridentate hydrazone and the monodentate pyridine ligands, with the N atoms in a *trans* arrangement about the Cu^II^ atom.

## Related literature

For the isostructural Ni complex, see: Zheng *et al.* (2009 ▶).
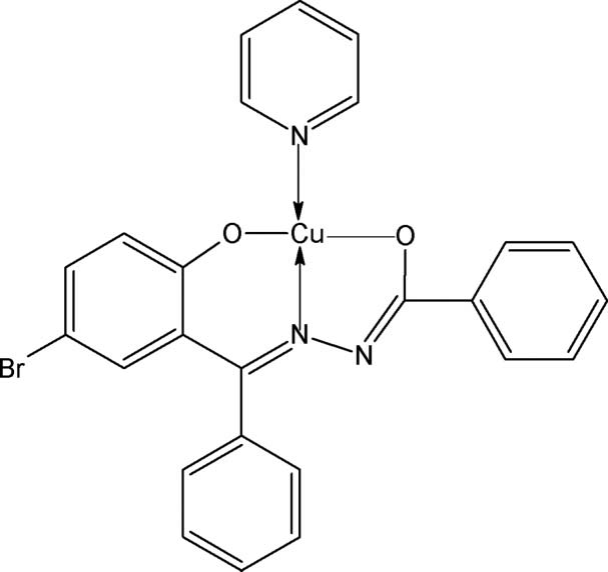


## Experimental

### 

#### Crystal data

[Cu(C_20_H_13_BrN_2_O_2_)(C_5_H_5_N)]
*M* *_r_* = 535.87
Monoclinic, 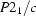

*a* = 22.655 (2) Å
*b* = 10.6514 (10) Å
*c* = 19.3388 (19) Å
β = 108.521 (2)°
*V* = 4424.9 (7) Å^3^
*Z* = 8
Mo *K*α radiation
μ = 2.82 mm^−1^
*T* = 295 K
0.16 × 0.12 × 0.08 mm

#### Data collection

Bruker APEXII CCD area-detector diffractometer
Absorption correction: multi-scan (*SADABS*; Bruker, 2005 ▶) *T*_min_ = 0.661, *T*_max_ = 0.806
22878 measured reflections
7831 independent reflections
5740 reflections with *I* > 2σ(*I*)
*R*_int_ = 0.029

#### Refinement

*R*[*F* ^2^ > 2σ(*F* ^2^)] = 0.034
*wR*(*F* ^2^) = 0.100
*S* = 1.09
7831 reflections
577 parameters
H-atom parameters constrained
Δρ_max_ = 0.46 e Å^−3^
Δρ_min_ = −0.57 e Å^−3^

### 

Data collection: *APEX2* (Bruker, 2005 ▶); cell refinement: *SAINT* (Bruker, 2005 ▶); data reduction: *SAINT*; program(s) used to solve structure: *SHELXS97* (Sheldrick, 2008 ▶); program(s) used to refine structure: *SHELXL97* (Sheldrick, 2008 ▶); molecular graphics: *SHELXTL* (Sheldrick, 2008 ▶); software used to prepare material for publication: *SHELXTL*.

## Supplementary Material

Crystal structure: contains datablocks I, global. DOI: 10.1107/S1600536809030463/rk2158sup1.cif

Structure factors: contains datablocks I. DOI: 10.1107/S1600536809030463/rk2158Isup2.hkl

Additional supplementary materials:  crystallographic information; 3D view; checkCIF report

## Figures and Tables

**Fig. 1.**
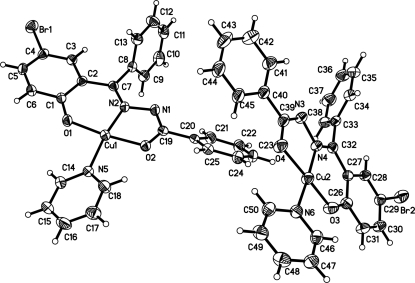
The asymmetric unit of title compound with the atom-numbering scheme. Displacement ellipsoids are drawn at the 30% probability level. H atoms are presented as a small spheres of arbitrary radius.

## supplementary crystallographic information

### Comment

This structure was solved in continue the investigation of complexes of transition metals (Ni) with ligands {*N*-[(*E*)-(5-bromo-2- -hydroxyphenyl)(phenyl)methylene]benzohydrazide}pyridinenickel(II) (Zheng *et al.*, 2009). These Ni-  and Cu-complexes (Fig.1) are isostructural. All bond distances and bond angles have a normal values.

### Experimental

A *DMF* solution (5 ml) of *N*-[(*E*)-(5-bromo-2- hydroxyphenyl)(phenyl)methylene]benzohydrazide (0.25 mmol, 0.099 g) was mixed with a methanol solution(5 ml) of CuCl_2_^.^2H_2_O (0.25 mmol, 0.043 g). The mixture was stirred at 298 K for 4 h and then filtered. A blue precipitate was produced after about 10 d. A pyridine mixture (5 ml) was used to dissolve the precipitate at 330 K. Blue block-shaped crystals of were obtained after one month (yield 30%).

### Refinement

All H atoms were placed geometrically at ideal positions and allowed to ride on the parent C atoms, with C–H = 0.93 Å and *U*_iso_(H) = 1.2*U*_eq_(C).

### Crystal data

**Table:**

[Cu(C_20_H_13_BrN_2_O_2_)(C_5_H_5_N)]	*F*(000) = 2152
*M**_r_* = 535.87	*D*_x_ = 1.609 Mg m^−^^3^
Monoclinic, *P*2_1_/*c*	Melting point: 330 K
Hall symbol: -P 2ybc	Mo *K*α radiation, λ = 0.71073 Å
*a* = 22.655 (2) Å	Cell parameters from 5857 reflections
*b* = 10.6514 (10) Å	θ = 2.2–26.2°
*c* = 19.3388 (19) Å	µ = 2.82 mm^−^^1^
β = 108.521 (2)°	*T* = 295 K
*V* = 4424.9 (7) Å^3^	Block, blue
*Z* = 8	0.16 × 0.12 × 0.08 mm

### Data collection

**Table:**

Bruker APEXII CCD area-detector diffractometer	7831 independent reflections
Radiation source: fine-focus sealed tube	5740 reflections with *I* > 2σ(*I*)
graphite	*R*_int_ = 0.029
φ and ω scans	θ_max_ = 25.1°, θ_min_ = 1.0°
Absorption correction: multi-scan (*SADABS*; Bruker, 2005)	*h* = −26→26
*T*_min_ = 0.661, *T*_max_ = 0.806	*k* = −12→12
22878 measured reflections	*l* = −23→18

### Refinement

**Table:**

Refinement on *F*^2^	Primary atom site location: structure-invariant direct methods
Least-squares matrix: full	Secondary atom site location: difference Fourier map
*R*[*F*^2^ > 2σ(*F*^2^)] = 0.034	Hydrogen site location: inferred from neighbouring sites
*wR*(*F*^2^) = 0.100	H-atom parameters constrained
*S* = 1.09	*w* = 1/[σ^2^(*F*_o_^2^) + (0.0541*P*)^2^] where *P* = (*F*_o_^2^ + 2*F*_c_^2^)/3
7831 reflections	(Δ/σ)_max_ = 0.001
577 parameters	Δρ_max_ = 0.46 e Å^−^^3^
0 restraints	Δρ_min_ = −0.57 e Å^−^^3^

### Special details

**Table:**

Geometry. All s.u.'s (except the s.u. in the dihedral angle between two l.s. planes) are estimated using the full covariance matrix. The cell s.u.'s are taken into account individually in the estimation of s.u.'s in distances, angles and torsion angles; correlations between s.u.'s in cell parameters are only used when they are defined by crystal symmetry. An approximate (isotropic) treatment of cell s.u.'s is used for estimating s.u.'s involving l.s. planes.
Refinement. Refinement of *F*^2^ against ALL reflections. The weighted *R*-factor *wR* and goodness of fit *S* are based on *F*^2^, conventional *R*-factors *R* are based on *F*, with *F* set to zero for negative *F*^2^. The threshold expression of *F*^2^ > σ(*F*^2^) is used only for calculating *R*-factors(gt) *etc*. and is not relevant to the choice of reflections for refinement. *R*-factors based on *F*^2^ are statistically about twice as large as those based on *F*, and *R*-factors based on ALL data will be even larger.

### Fractional atomic coordinates and isotropic or equivalent isotropic displacement parameters (Å^2^)

**Table:**

	*x*	*y*	*z*	*U*_iso_*/*U*_eq_	
Cu1	0.448059 (18)	0.58286 (3)	0.36047 (2)	0.04049 (12)	
Cu2	0.06456 (2)	0.19326 (4)	0.09827 (2)	0.04655 (13)	
Br1	0.756186 (18)	0.42943 (4)	0.61827 (2)	0.06374 (14)	
Br2	0.08695 (2)	−0.23047 (4)	−0.19252 (2)	0.06763 (14)	
O1	0.51513 (10)	0.65304 (19)	0.43260 (13)	0.0470 (6)	
O2	0.37982 (10)	0.4965 (2)	0.29422 (12)	0.0442 (5)	
O3	0.03949 (12)	0.1747 (2)	−0.00216 (13)	0.0574 (7)	
O4	0.09937 (12)	0.2097 (2)	0.20165 (13)	0.0561 (6)	
N1	0.44828 (13)	0.3300 (2)	0.32130 (15)	0.0437 (7)	
N2	0.48543 (12)	0.4188 (2)	0.36919 (14)	0.0386 (6)	
N3	0.14631 (13)	0.0215 (3)	0.18986 (14)	0.0469 (7)	
N4	0.11394 (12)	0.0421 (3)	0.11583 (14)	0.0423 (7)	
N5	0.40778 (13)	0.7504 (2)	0.33205 (15)	0.0424 (7)	
N6	0.01464 (13)	0.3501 (3)	0.08826 (16)	0.0467 (7)	
C1	0.56516 (15)	0.5958 (3)	0.47237 (17)	0.0364 (7)	
C2	0.57943 (14)	0.4665 (3)	0.46661 (17)	0.0376 (7)	
C3	0.63679 (15)	0.4215 (3)	0.51111 (18)	0.0417 (8)	
H3	0.6471	0.3381	0.5066	0.050*	
C4	0.67815 (14)	0.4958 (3)	0.56095 (18)	0.0433 (8)	
C5	0.66359 (17)	0.6198 (3)	0.56923 (19)	0.0475 (9)	
H5	0.6912	0.6703	0.6039	0.057*	
C6	0.60845 (16)	0.6672 (3)	0.52612 (19)	0.0449 (8)	
H6	0.5990	0.7505	0.5325	0.054*	
C7	0.53780 (15)	0.3801 (3)	0.41444 (18)	0.0375 (7)	
C8	0.55608 (14)	0.2454 (3)	0.41456 (18)	0.0373 (8)	
C9	0.57715 (17)	0.1986 (3)	0.3606 (2)	0.0530 (9)	
H9	0.5779	0.2498	0.3219	0.064*	
C10	0.59715 (18)	0.0761 (4)	0.3635 (2)	0.0638 (11)	
H10	0.6121	0.0455	0.3271	0.077*	
C11	0.59528 (18)	−0.0013 (4)	0.4196 (2)	0.0613 (11)	
H11	0.6090	−0.0840	0.4215	0.074*	
C12	0.5731 (2)	0.0446 (3)	0.4724 (2)	0.0658 (11)	
H12	0.5711	−0.0074	0.5102	0.079*	
C13	0.55368 (17)	0.1672 (3)	0.4700 (2)	0.0507 (9)	
H13	0.5388	0.1975	0.5064	0.061*	
C14	0.44054 (18)	0.8571 (3)	0.3428 (2)	0.0542 (10)	
H14	0.4836	0.8525	0.3629	0.065*	
C15	0.4137 (2)	0.9730 (3)	0.3254 (2)	0.0645 (11)	
H15	0.4380	1.0453	0.3342	0.077*	
C16	0.3504 (2)	0.9803 (4)	0.2948 (2)	0.0681 (12)	
H16	0.3308	1.0579	0.2835	0.082*	
C17	0.31676 (19)	0.8725 (4)	0.2812 (2)	0.0644 (11)	
H17	0.2739	0.8753	0.2586	0.077*	
C18	0.34619 (17)	0.7597 (3)	0.3008 (2)	0.0539 (10)	
H18	0.3224	0.6867	0.2921	0.065*	
C19	0.39589 (15)	0.3824 (3)	0.28524 (18)	0.0390 (8)	
C20	0.35050 (15)	0.3070 (3)	0.22877 (18)	0.0396 (8)	
C21	0.36878 (18)	0.2208 (3)	0.1867 (2)	0.0511 (9)	
H21	0.4109	0.2065	0.1947	0.061*	
C22	0.3249 (2)	0.1554 (4)	0.1325 (2)	0.0626 (11)	
H22	0.3375	0.0977	0.1040	0.075*	
C23	0.2631 (2)	0.1753 (4)	0.1210 (2)	0.0632 (11)	
H23	0.2337	0.1313	0.0844	0.076*	
C24	0.24391 (18)	0.2598 (4)	0.1628 (2)	0.0616 (11)	
H24	0.2017	0.2716	0.1553	0.074*	
C25	0.28738 (16)	0.3275 (3)	0.21613 (19)	0.0482 (9)	
H25	0.2744	0.3868	0.2435	0.058*	
C26	0.05294 (16)	0.0824 (3)	−0.03941 (18)	0.0444 (8)	
C27	0.09257 (14)	−0.0200 (3)	−0.00941 (17)	0.0396 (8)	
C28	0.10089 (16)	−0.1110 (3)	−0.05787 (18)	0.0431 (8)	
H28	0.1266	−0.1794	−0.0394	0.052*	
C29	0.07243 (16)	−0.1022 (3)	−0.13131 (18)	0.0448 (8)	
C30	0.03406 (17)	−0.0032 (3)	−0.16105 (19)	0.0511 (9)	
H30	0.0146	0.0021	−0.2111	0.061*	
C31	0.02517 (17)	0.0870 (3)	−0.11555 (19)	0.0523 (9)	
H31	−0.0004	0.1548	−0.1357	0.063*	
C32	0.12314 (15)	−0.0372 (3)	0.06895 (18)	0.0411 (8)	
C33	0.16432 (15)	−0.1486 (3)	0.09448 (17)	0.0422 (8)	
C34	0.14936 (18)	−0.2398 (3)	0.1365 (2)	0.0545 (10)	
H34	0.1143	−0.2306	0.1511	0.065*	
C35	0.1865 (2)	−0.3456 (4)	0.1570 (2)	0.0683 (12)	
H35	0.1760	−0.4074	0.1850	0.082*	
C36	0.2384 (2)	−0.3594 (4)	0.1361 (2)	0.0724 (12)	
H36	0.2632	−0.4306	0.1498	0.087*	
C37	0.2538 (2)	−0.2684 (4)	0.0954 (2)	0.0683 (12)	
H37	0.2891	−0.2776	0.0812	0.082*	
C38	0.21764 (17)	−0.1639 (3)	0.0751 (2)	0.0530 (9)	
H38	0.2290	−0.1020	0.0479	0.064*	
C39	0.13458 (16)	0.1139 (4)	0.22802 (19)	0.0472 (9)	
C40	0.16633 (16)	0.1121 (4)	0.30833 (19)	0.0492 (9)	
C41	0.19493 (19)	0.0057 (4)	0.3430 (2)	0.0632 (11)	
H41	0.1939	−0.0674	0.3163	0.076*	
C42	0.2255 (2)	0.0061 (5)	0.4175 (2)	0.0819 (14)	
H42	0.2447	−0.0665	0.4409	0.098*	
C43	0.2271 (2)	0.1147 (5)	0.4568 (2)	0.0819 (14)	
H43	0.2479	0.1161	0.5067	0.098*	
C44	0.1983 (2)	0.2194 (5)	0.4226 (2)	0.0745 (13)	
H44	0.1994	0.2923	0.4494	0.089*	
C45	0.16749 (18)	0.2197 (4)	0.3487 (2)	0.0610 (10)	
H45	0.1475	0.2921	0.3260	0.073*	
C46	−0.03592 (16)	0.3678 (3)	0.0300 (2)	0.0505 (9)	
H46	−0.0454	0.3083	−0.0072	0.061*	
C47	−0.07398 (18)	0.4696 (4)	0.0230 (2)	0.0573 (10)	
H47	−0.1095	0.4774	−0.0173	0.069*	
C48	−0.0591 (2)	0.5604 (4)	0.0763 (2)	0.0661 (11)	
H48	−0.0840	0.6312	0.0725	0.079*	
C49	−0.0072 (2)	0.5442 (4)	0.1347 (2)	0.0663 (12)	
H49	0.0043	0.6051	0.1710	0.080*	
C50	0.02816 (19)	0.4389 (3)	0.1403 (2)	0.0587 (10)	
H50	0.0628	0.4282	0.1814	0.070*	

### Atomic displacement parameters (Å^2^)

**Table:**

	*U*^11^	*U*^22^	*U*^33^	*U*^12^	*U*^13^	*U*^23^
Cu1	0.0447 (2)	0.0330 (2)	0.0410 (2)	−0.00132 (18)	0.00960 (19)	−0.00144 (18)
Cu2	0.0528 (3)	0.0488 (3)	0.0367 (2)	0.0020 (2)	0.0122 (2)	−0.0052 (2)
Br1	0.0474 (2)	0.0744 (3)	0.0589 (3)	0.00269 (19)	0.00196 (19)	0.0008 (2)
Br2	0.0839 (3)	0.0733 (3)	0.0418 (2)	0.0172 (2)	0.0144 (2)	−0.0132 (2)
O1	0.0473 (14)	0.0341 (12)	0.0517 (14)	0.0019 (11)	0.0044 (12)	−0.0080 (11)
O2	0.0460 (13)	0.0347 (12)	0.0447 (13)	0.0008 (10)	0.0042 (11)	−0.0020 (11)
O3	0.0793 (18)	0.0525 (15)	0.0384 (14)	0.0205 (13)	0.0158 (13)	−0.0018 (12)
O4	0.0665 (17)	0.0606 (16)	0.0377 (14)	0.0078 (14)	0.0118 (12)	−0.0078 (12)
N1	0.0463 (17)	0.0344 (14)	0.0448 (17)	−0.0022 (13)	0.0065 (14)	−0.0063 (13)
N2	0.0412 (16)	0.0334 (14)	0.0377 (15)	−0.0024 (13)	0.0075 (13)	−0.0035 (13)
N3	0.0518 (18)	0.0538 (18)	0.0311 (15)	−0.0030 (14)	0.0073 (13)	−0.0025 (14)
N4	0.0470 (17)	0.0462 (16)	0.0315 (15)	−0.0017 (13)	0.0096 (13)	0.0008 (13)
N5	0.0488 (18)	0.0361 (15)	0.0393 (16)	−0.0018 (13)	0.0099 (14)	0.0008 (13)
N6	0.0538 (18)	0.0469 (17)	0.0410 (17)	−0.0006 (14)	0.0174 (15)	−0.0050 (14)
C1	0.0395 (19)	0.0353 (17)	0.0364 (18)	−0.0045 (15)	0.0149 (15)	−0.0033 (15)
C2	0.0386 (18)	0.0414 (18)	0.0334 (17)	−0.0016 (15)	0.0125 (15)	−0.0003 (15)
C3	0.044 (2)	0.0413 (18)	0.0403 (19)	−0.0010 (16)	0.0143 (16)	−0.0009 (16)
C4	0.0352 (18)	0.055 (2)	0.0380 (19)	−0.0030 (16)	0.0089 (15)	0.0013 (17)
C5	0.052 (2)	0.048 (2)	0.040 (2)	−0.0154 (17)	0.0117 (17)	−0.0066 (17)
C6	0.053 (2)	0.0357 (18)	0.045 (2)	−0.0033 (16)	0.0144 (18)	−0.0049 (17)
C7	0.0431 (19)	0.0351 (17)	0.0372 (18)	−0.0024 (15)	0.0171 (16)	0.0001 (15)
C8	0.0343 (18)	0.0387 (18)	0.0377 (19)	−0.0014 (14)	0.0096 (15)	−0.0062 (15)
C9	0.061 (2)	0.055 (2)	0.045 (2)	−0.0011 (19)	0.0192 (19)	−0.0056 (19)
C10	0.065 (3)	0.063 (3)	0.069 (3)	0.002 (2)	0.031 (2)	−0.028 (2)
C11	0.060 (3)	0.038 (2)	0.080 (3)	0.0075 (18)	0.014 (2)	−0.016 (2)
C12	0.091 (3)	0.042 (2)	0.070 (3)	0.009 (2)	0.033 (2)	0.005 (2)
C13	0.068 (2)	0.041 (2)	0.048 (2)	0.0050 (18)	0.0250 (19)	−0.0056 (18)
C14	0.057 (2)	0.042 (2)	0.055 (2)	−0.0080 (18)	0.0061 (19)	−0.0007 (18)
C15	0.084 (3)	0.036 (2)	0.065 (3)	−0.005 (2)	0.012 (2)	0.005 (2)
C16	0.089 (3)	0.042 (2)	0.073 (3)	0.013 (2)	0.027 (3)	0.013 (2)
C17	0.060 (3)	0.060 (3)	0.073 (3)	0.013 (2)	0.020 (2)	0.007 (2)
C18	0.051 (2)	0.044 (2)	0.065 (3)	−0.0008 (18)	0.016 (2)	0.0023 (19)
C19	0.043 (2)	0.0373 (18)	0.0363 (18)	−0.0040 (15)	0.0126 (16)	0.0039 (15)
C20	0.048 (2)	0.0332 (17)	0.0360 (18)	−0.0062 (15)	0.0105 (15)	0.0059 (15)
C21	0.058 (2)	0.047 (2)	0.051 (2)	−0.0079 (18)	0.0209 (19)	−0.0020 (18)
C22	0.089 (3)	0.056 (2)	0.045 (2)	−0.018 (2)	0.024 (2)	−0.011 (2)
C23	0.076 (3)	0.057 (2)	0.045 (2)	−0.028 (2)	0.003 (2)	−0.001 (2)
C24	0.047 (2)	0.059 (2)	0.068 (3)	−0.0107 (19)	0.003 (2)	0.007 (2)
C25	0.052 (2)	0.0404 (19)	0.048 (2)	−0.0026 (17)	0.0108 (18)	0.0028 (17)
C26	0.053 (2)	0.046 (2)	0.0371 (19)	0.0000 (17)	0.0172 (17)	−0.0018 (17)
C27	0.044 (2)	0.0424 (19)	0.0334 (18)	−0.0050 (16)	0.0133 (15)	0.0017 (16)
C28	0.049 (2)	0.0423 (19)	0.0366 (19)	0.0045 (16)	0.0113 (16)	0.0001 (16)
C29	0.053 (2)	0.049 (2)	0.0333 (18)	0.0008 (17)	0.0139 (16)	−0.0054 (16)
C30	0.059 (2)	0.062 (2)	0.0304 (19)	0.0053 (19)	0.0116 (17)	−0.0001 (18)
C31	0.060 (2)	0.054 (2)	0.039 (2)	0.0149 (19)	0.0110 (18)	0.0039 (18)
C32	0.044 (2)	0.0421 (18)	0.0367 (19)	−0.0042 (16)	0.0123 (16)	−0.0025 (16)
C33	0.049 (2)	0.0428 (19)	0.0301 (18)	−0.0017 (16)	0.0056 (16)	−0.0021 (16)
C34	0.057 (2)	0.057 (2)	0.045 (2)	−0.0047 (19)	0.0089 (18)	0.0064 (19)
C35	0.084 (3)	0.055 (3)	0.053 (3)	−0.003 (2)	0.005 (2)	0.013 (2)
C36	0.073 (3)	0.058 (3)	0.069 (3)	0.017 (2)	−0.002 (2)	0.011 (2)
C37	0.060 (3)	0.072 (3)	0.064 (3)	0.018 (2)	0.009 (2)	0.002 (2)
C38	0.057 (2)	0.053 (2)	0.046 (2)	0.0052 (19)	0.0114 (18)	0.0077 (18)
C39	0.046 (2)	0.059 (2)	0.036 (2)	−0.0102 (18)	0.0132 (17)	−0.0064 (18)
C40	0.049 (2)	0.061 (2)	0.037 (2)	−0.0136 (18)	0.0136 (17)	−0.0046 (19)
C41	0.070 (3)	0.072 (3)	0.045 (2)	−0.002 (2)	0.016 (2)	−0.002 (2)
C42	0.086 (3)	0.100 (4)	0.051 (3)	0.006 (3)	0.009 (2)	0.005 (3)
C43	0.073 (3)	0.124 (4)	0.039 (2)	−0.017 (3)	0.005 (2)	−0.010 (3)
C44	0.087 (3)	0.086 (3)	0.047 (3)	−0.027 (3)	0.017 (2)	−0.018 (3)
C45	0.066 (3)	0.070 (3)	0.043 (2)	−0.019 (2)	0.0124 (19)	−0.009 (2)
C46	0.051 (2)	0.054 (2)	0.047 (2)	−0.0056 (19)	0.0173 (19)	−0.0084 (19)
C47	0.051 (2)	0.062 (2)	0.057 (2)	0.007 (2)	0.0148 (19)	0.002 (2)
C48	0.086 (3)	0.056 (2)	0.065 (3)	0.014 (2)	0.036 (3)	0.004 (2)
C49	0.105 (4)	0.046 (2)	0.049 (2)	0.004 (2)	0.027 (3)	−0.0089 (19)
C50	0.076 (3)	0.058 (2)	0.039 (2)	−0.004 (2)	0.0144 (19)	−0.0071 (19)

### Geometric parameters (Å, °)

**Table:**

Cu1—O1	1.862 (2)	C19—C20	1.477 (4)
Cu1—O2	1.903 (2)	C20—C21	1.375 (5)
Cu1—N2	1.925 (2)	C20—C25	1.389 (5)
Cu1—N5	2.000 (3)	C21—C22	1.382 (5)
Cu2—O3	1.853 (2)	C21—H21	0.9300
Cu2—O4	1.910 (2)	C22—C23	1.363 (6)
Cu2—N4	1.928 (3)	C22—H22	0.9300
Cu2—N6	1.993 (3)	C23—C24	1.370 (6)
Br1—C4	1.900 (3)	C23—H23	0.9300
Br2—C29	1.903 (3)	C24—C25	1.381 (5)
O1—C1	1.302 (4)	C24—H24	0.9300
O2—C19	1.296 (4)	C25—H25	0.9300
O3—C26	1.310 (4)	C26—C31	1.406 (5)
O4—C39	1.296 (4)	C26—C27	1.415 (4)
N1—C19	1.296 (4)	C27—C28	1.402 (4)
N1—N2	1.402 (3)	C27—C32	1.464 (4)
N2—C7	1.298 (4)	C28—C29	1.364 (5)
N3—C39	1.307 (4)	C28—H28	0.9300
N3—N4	1.403 (4)	C29—C30	1.371 (5)
N4—C32	1.304 (4)	C30—C31	1.361 (5)
N5—C18	1.337 (4)	C30—H30	0.9300
N5—C14	1.338 (4)	C31—H31	0.9300
N6—C46	1.341 (4)	C32—C33	1.493 (5)
N6—C50	1.343 (4)	C33—C34	1.376 (5)
C1—C6	1.405 (4)	C33—C38	1.383 (5)
C1—C2	1.427 (4)	C34—C35	1.386 (5)
C2—C3	1.396 (4)	C34—H34	0.9300
C2—C7	1.466 (4)	C35—C36	1.368 (6)
C3—C4	1.364 (4)	C35—H35	0.9300
C3—H3	0.9300	C36—C37	1.362 (6)
C4—C5	1.382 (5)	C36—H36	0.9300
C5—C6	1.360 (5)	C37—C38	1.363 (5)
C5—H5	0.9300	C37—H37	0.9300
C6—H6	0.9300	C38—H38	0.9300
C7—C8	1.493 (4)	C39—C40	1.490 (5)
C8—C9	1.372 (5)	C40—C41	1.370 (5)
C8—C13	1.373 (5)	C40—C45	1.382 (5)
C9—C10	1.376 (5)	C41—C42	1.387 (6)
C9—H9	0.9300	C41—H41	0.9300
C10—C11	1.374 (6)	C42—C43	1.378 (6)
C10—H10	0.9300	C42—H42	0.9300
C11—C12	1.364 (5)	C43—C44	1.353 (6)
C11—H11	0.9300	C43—H43	0.9300
C12—C13	1.374 (5)	C44—C45	1.376 (6)
C12—H12	0.9300	C44—H44	0.9300
C13—H13	0.9300	C45—H45	0.9300
C14—C15	1.370 (5)	C46—C47	1.365 (5)
C14—H14	0.9300	C46—H46	0.9300
C15—C16	1.367 (5)	C47—C48	1.376 (5)
C15—H15	0.9300	C47—H47	0.9300
C16—C17	1.358 (5)	C48—C49	1.358 (6)
C16—H16	0.9300	C48—H48	0.9300
C17—C18	1.368 (5)	C49—C50	1.363 (5)
C17—H17	0.9300	C49—H49	0.9300
C18—H18	0.9300	C50—H50	0.9300
			
O1—Cu1—O2	173.64 (10)	C20—C21—C22	120.4 (4)
O1—Cu1—N2	93.85 (10)	C20—C21—H21	119.8
O2—Cu1—N2	82.00 (10)	C22—C21—H21	119.8
O1—Cu1—N5	92.31 (10)	C23—C22—C21	120.0 (4)
O2—Cu1—N5	92.53 (10)	C23—C22—H22	120.0
N2—Cu1—N5	169.48 (11)	C21—C22—H22	120.0
O3—Cu2—O4	173.82 (11)	C22—C23—C24	120.5 (4)
O3—Cu2—N4	93.61 (11)	C22—C23—H23	119.8
O4—Cu2—N4	82.44 (11)	C24—C23—H23	119.8
O3—Cu2—N6	90.70 (11)	C23—C24—C25	119.9 (4)
O4—Cu2—N6	93.32 (11)	C23—C24—H24	120.0
N4—Cu2—N6	175.65 (12)	C25—C24—H24	120.0
C1—O1—Cu1	127.20 (19)	C24—C25—C20	120.0 (4)
C19—O2—Cu1	109.9 (2)	C24—C25—H25	120.0
C26—O3—Cu2	127.5 (2)	C20—C25—H25	120.0
C39—O4—Cu2	109.7 (2)	O3—C26—C31	116.4 (3)
C19—N1—N2	108.7 (3)	O3—C26—C27	125.5 (3)
C7—N2—N1	117.4 (3)	C31—C26—C27	118.1 (3)
C7—N2—Cu1	129.0 (2)	C28—C27—C26	117.5 (3)
N1—N2—Cu1	113.54 (19)	C28—C27—C32	119.0 (3)
C39—N3—N4	108.9 (3)	C26—C27—C32	123.4 (3)
C32—N4—N3	117.5 (3)	C29—C28—C27	122.0 (3)
C32—N4—Cu2	129.1 (2)	C29—C28—H28	119.0
N3—N4—Cu2	113.3 (2)	C27—C28—H28	119.0
C18—N5—C14	117.1 (3)	C28—C29—C30	121.1 (3)
C18—N5—Cu1	120.7 (2)	C28—C29—Br2	118.7 (3)
C14—N5—Cu1	122.3 (2)	C30—C29—Br2	120.2 (3)
C46—N6—C50	117.5 (3)	C31—C30—C29	118.5 (3)
C46—N6—Cu2	120.4 (2)	C31—C30—H30	120.8
C50—N6—Cu2	122.0 (3)	C29—C30—H30	120.8
O1—C1—C6	117.1 (3)	C30—C31—C26	122.9 (3)
O1—C1—C2	125.6 (3)	C30—C31—H31	118.5
C6—C1—C2	117.3 (3)	C26—C31—H31	118.5
C3—C2—C1	118.2 (3)	N4—C32—C27	120.7 (3)
C3—C2—C7	118.6 (3)	N4—C32—C33	120.4 (3)
C1—C2—C7	123.2 (3)	C27—C32—C33	118.8 (3)
C4—C3—C2	122.2 (3)	C34—C33—C38	118.4 (3)
C4—C3—H3	118.9	C34—C33—C32	120.7 (3)
C2—C3—H3	118.9	C38—C33—C32	120.8 (3)
C3—C4—C5	120.0 (3)	C33—C34—C35	120.2 (4)
C3—C4—Br1	119.9 (3)	C33—C34—H34	119.9
C5—C4—Br1	120.0 (3)	C35—C34—H34	119.9
C6—C5—C4	119.3 (3)	C36—C35—C34	120.2 (4)
C6—C5—H5	120.3	C36—C35—H35	119.9
C4—C5—H5	120.3	C34—C35—H35	119.9
C5—C6—C1	122.8 (3)	C37—C36—C35	119.7 (4)
C5—C6—H6	118.6	C37—C36—H36	120.1
C1—C6—H6	118.6	C35—C36—H36	120.1
N2—C7—C2	121.0 (3)	C36—C37—C38	120.4 (4)
N2—C7—C8	120.0 (3)	C36—C37—H37	119.8
C2—C7—C8	119.0 (3)	C38—C37—H37	119.8
C9—C8—C13	118.9 (3)	C37—C38—C33	121.0 (4)
C9—C8—C7	120.8 (3)	C37—C38—H38	119.5
C13—C8—C7	120.3 (3)	C33—C38—H38	119.5
C8—C9—C10	120.2 (4)	O4—C39—N3	125.5 (3)
C8—C9—H9	119.9	O4—C39—C40	117.0 (3)
C10—C9—H9	119.9	N3—C39—C40	117.5 (3)
C11—C10—C9	120.6 (4)	C41—C40—C45	119.2 (4)
C11—C10—H10	119.7	C41—C40—C39	121.1 (3)
C9—C10—H10	119.7	C45—C40—C39	119.6 (4)
C12—C11—C10	119.1 (3)	C40—C41—C42	120.6 (4)
C12—C11—H11	120.4	C40—C41—H41	119.7
C10—C11—H11	120.4	C42—C41—H41	119.7
C11—C12—C13	120.4 (4)	C43—C42—C41	119.5 (5)
C11—C12—H12	119.8	C43—C42—H42	120.3
C13—C12—H12	119.8	C41—C42—H42	120.3
C8—C13—C12	120.7 (3)	C44—C43—C42	119.9 (4)
C8—C13—H13	119.6	C44—C43—H43	120.1
C12—C13—H13	119.6	C42—C43—H43	120.1
N5—C14—C15	123.1 (4)	C43—C44—C45	121.1 (4)
N5—C14—H14	118.4	C43—C44—H44	119.4
C15—C14—H14	118.4	C45—C44—H44	119.4
C16—C15—C14	118.7 (4)	C44—C45—C40	119.7 (4)
C16—C15—H15	120.6	C44—C45—H45	120.1
C14—C15—H15	120.6	C40—C45—H45	120.1
C17—C16—C15	118.9 (4)	N6—C46—C47	122.6 (3)
C17—C16—H16	120.6	N6—C46—H46	118.7
C15—C16—H16	120.6	C47—C46—H46	118.7
C16—C17—C18	119.7 (4)	C46—C47—C48	119.2 (4)
C16—C17—H17	120.2	C46—C47—H47	120.4
C18—C17—H17	120.2	C48—C47—H47	120.4
N5—C18—C17	122.5 (3)	C49—C48—C47	118.4 (4)
N5—C18—H18	118.7	C49—C48—H48	120.8
C17—C18—H18	118.7	C47—C48—H48	120.8
O2—C19—N1	125.4 (3)	C48—C49—C50	120.1 (4)
O2—C19—C20	116.7 (3)	C48—C49—H49	119.9
N1—C19—C20	117.8 (3)	C50—C49—H49	119.9
C21—C20—C25	119.2 (3)	N6—C50—C49	122.2 (4)
C21—C20—C19	122.1 (3)	N6—C50—H50	118.9
C25—C20—C19	118.7 (3)	C49—C50—H50	118.9
			
N2—Cu1—O1—C1	−2.7 (3)	Cu1—O2—C19—N1	6.6 (4)
N5—Cu1—O1—C1	−174.2 (3)	Cu1—O2—C19—C20	−173.0 (2)
N2—Cu1—O2—C19	−5.8 (2)	N2—N1—C19—O2	−2.4 (4)
N5—Cu1—O2—C19	165.1 (2)	N2—N1—C19—C20	177.2 (3)
N4—Cu2—O3—C26	2.2 (3)	O2—C19—C20—C21	144.6 (3)
N6—Cu2—O3—C26	−177.1 (3)	N1—C19—C20—C21	−35.1 (5)
N4—Cu2—O4—C39	−1.7 (2)	O2—C19—C20—C25	−33.0 (4)
N6—Cu2—O4—C39	177.3 (2)	N1—C19—C20—C25	147.4 (3)
C19—N1—N2—C7	174.3 (3)	C25—C20—C21—C22	−0.1 (5)
C19—N1—N2—Cu1	−3.0 (3)	C19—C20—C21—C22	−177.6 (3)
O1—Cu1—N2—C7	3.3 (3)	C20—C21—C22—C23	−0.5 (6)
O2—Cu1—N2—C7	−171.9 (3)	C21—C22—C23—C24	−0.2 (6)
N5—Cu1—N2—C7	129.0 (6)	C22—C23—C24—C25	1.4 (6)
O1—Cu1—N2—N1	−179.8 (2)	C23—C24—C25—C20	−2.0 (5)
O2—Cu1—N2—N1	5.0 (2)	C21—C20—C25—C24	1.3 (5)
N5—Cu1—N2—N1	−54.1 (7)	C19—C20—C25—C24	178.9 (3)
C39—N3—N4—C32	175.9 (3)	Cu2—O3—C26—C31	176.6 (2)
C39—N3—N4—Cu2	−0.9 (3)	Cu2—O3—C26—C27	−3.3 (5)
O3—Cu2—N4—C32	0.3 (3)	O3—C26—C27—C28	179.2 (3)
O4—Cu2—N4—C32	−174.9 (3)	C31—C26—C27—C28	−0.7 (5)
O3—Cu2—N4—N3	176.7 (2)	O3—C26—C27—C32	1.3 (5)
O4—Cu2—N4—N3	1.5 (2)	C31—C26—C27—C32	−178.6 (3)
O1—Cu1—N5—C18	−154.3 (3)	C26—C27—C28—C29	0.3 (5)
O2—Cu1—N5—C18	21.5 (3)	C32—C27—C28—C29	178.3 (3)
N2—Cu1—N5—C18	79.8 (7)	C27—C28—C29—C30	−0.1 (5)
O1—Cu1—N5—C14	26.0 (3)	C27—C28—C29—Br2	179.8 (2)
O2—Cu1—N5—C14	−158.1 (3)	C28—C29—C30—C31	0.4 (6)
N2—Cu1—N5—C14	−99.8 (6)	Br2—C29—C30—C31	−179.6 (3)
O3—Cu2—N6—C46	27.0 (3)	C29—C30—C31—C26	−0.8 (6)
O4—Cu2—N6—C46	−157.7 (3)	O3—C26—C31—C30	−178.9 (3)
O3—Cu2—N6—C50	−156.0 (3)	C27—C26—C31—C30	1.0 (5)
O4—Cu2—N6—C50	19.3 (3)	N3—N4—C32—C27	−178.1 (3)
Cu1—O1—C1—C6	−177.0 (2)	Cu2—N4—C32—C27	−1.9 (5)
Cu1—O1—C1—C2	2.2 (5)	N3—N4—C32—C33	2.9 (4)
O1—C1—C2—C3	176.7 (3)	Cu2—N4—C32—C33	179.1 (2)
C6—C1—C2—C3	−4.0 (4)	C28—C27—C32—N4	−176.5 (3)
O1—C1—C2—C7	−0.9 (5)	C26—C27—C32—N4	1.3 (5)
C6—C1—C2—C7	178.3 (3)	C28—C27—C32—C33	2.5 (4)
C1—C2—C3—C4	2.2 (5)	C26—C27—C32—C33	−179.7 (3)
C7—C2—C3—C4	179.9 (3)	N4—C32—C33—C34	63.1 (4)
C2—C3—C4—C5	0.7 (5)	C27—C32—C33—C34	−115.9 (4)
C2—C3—C4—Br1	−178.6 (2)	N4—C32—C33—C38	−118.3 (4)
C3—C4—C5—C6	−1.5 (5)	C27—C32—C33—C38	62.7 (4)
Br1—C4—C5—C6	177.8 (3)	C38—C33—C34—C35	−1.5 (5)
C4—C5—C6—C1	−0.6 (5)	C32—C33—C34—C35	177.0 (3)
O1—C1—C6—C5	−177.3 (3)	C33—C34—C35—C36	0.5 (6)
C2—C1—C6—C5	3.4 (5)	C34—C35—C36—C37	0.3 (7)
N1—N2—C7—C2	−179.8 (3)	C35—C36—C37—C38	−0.1 (7)
Cu1—N2—C7—C2	−3.0 (4)	C36—C37—C38—C33	−1.0 (6)
N1—N2—C7—C8	−0.6 (4)	C34—C33—C38—C37	1.8 (5)
Cu1—N2—C7—C8	176.2 (2)	C32—C33—C38—C37	−176.8 (3)
C3—C2—C7—N2	−176.3 (3)	Cu2—O4—C39—N3	1.9 (4)
C1—C2—C7—N2	1.3 (5)	Cu2—O4—C39—C40	179.3 (2)
C3—C2—C7—C8	4.5 (4)	N4—N3—C39—O4	−0.7 (5)
C1—C2—C7—C8	−177.9 (3)	N4—N3—C39—C40	−178.1 (3)
N2—C7—C8—C9	75.3 (4)	O4—C39—C40—C41	167.8 (3)
C2—C7—C8—C9	−105.5 (4)	N3—C39—C40—C41	−14.7 (5)
N2—C7—C8—C13	−106.5 (4)	O4—C39—C40—C45	−12.7 (5)
C2—C7—C8—C13	72.7 (4)	N3—C39—C40—C45	164.8 (3)
C13—C8—C9—C10	−1.8 (5)	C45—C40—C41—C42	−0.9 (6)
C7—C8—C9—C10	176.3 (3)	C39—C40—C41—C42	178.6 (4)
C8—C9—C10—C11	1.2 (6)	C40—C41—C42—C43	−0.2 (7)
C9—C10—C11—C12	0.2 (6)	C41—C42—C43—C44	0.8 (7)
C10—C11—C12—C13	−0.9 (6)	C42—C43—C44—C45	−0.3 (7)
C9—C8—C13—C12	1.2 (5)	C43—C44—C45—C40	−0.8 (7)
C7—C8—C13—C12	−177.0 (3)	C41—C40—C45—C44	1.4 (6)
C11—C12—C13—C8	0.2 (6)	C39—C40—C45—C44	−178.1 (3)
C18—N5—C14—C15	2.1 (5)	C50—N6—C46—C47	−1.6 (5)
Cu1—N5—C14—C15	−178.3 (3)	Cu2—N6—C46—C47	175.5 (3)
N5—C14—C15—C16	−0.8 (6)	N6—C46—C47—C48	2.4 (6)
C14—C15—C16—C17	−1.5 (6)	C46—C47—C48—C49	−0.9 (6)
C15—C16—C17—C18	2.6 (6)	C47—C48—C49—C50	−1.2 (6)
C14—N5—C18—C17	−1.0 (6)	C46—N6—C50—C49	−0.6 (5)
Cu1—N5—C18—C17	179.4 (3)	Cu2—N6—C50—C49	−177.6 (3)
C16—C17—C18—N5	−1.3 (6)	C48—C49—C50—N6	2.0 (6)
